# Peritoneal Dialysis-Related Peritonitis Caused by Staphylococcus caprae: A Case Report

**DOI:** 10.7759/cureus.88870

**Published:** 2025-07-27

**Authors:** Mayu Iwata, Kenta Torigoe, Sayaka Honda, Rei Ono, Reira Takao, Mineaki Kitamura, Takahiro Takazono, Noriho Sakamoto, Kumiko Muta, Hiroshi Mukae, Tomoya Nishino

**Affiliations:** 1 Department of Nephrology, Nagasaki University Hospital, Nagasaki, JPN; 2 Department of Respiratory Medicine, Nagasaki University Graduate School of Biomedical Sciences, Nagasaki, JPN

**Keywords:** antibiotic therapy, end-stage renal disease (esrd), peritoneal dialysis, peritonitis, staphylococcus caprae

## Abstract

Herein, we report a case of peritoneal dialysis (PD)-related peritonitis caused by *Staphylococcus caprae* (*S. caprae*). An 88-year-old man who had been receiving PD presented with cloudy effluent and an elevated dialysate white blood cell (WBC) count of 800/μL. He was diagnosed with PD-related peritonitis and treated with intraperitoneal cefazolin and ceftazidime, which promptly cleared the effluent by day 3 and reduced the WBC count in the dialysate to 0/μL by day 5. Culturing of the effluent identified *S. caprae* (methicillin-resistant coagulase-negative *Staphylococcus*), so the antibiotic regimen was switched to intraperitoneal vancomycin. The infection resolved without recurrence, and the patient was discharged on day 17 of his hospitalization without requiring catheter removal. Although *S. caprae* has been implicated in infections of various organs, reports of PD-related peritonitis caused by this bacterium are rare in the literature. This case suggests that *S. caprae*-related PD peritonitis responds well to antibiotic therapy with intraperitoneal vancomycin and that patients with this infection have a generally favorable prognosis.

## Introduction

Peritoneal dialysis (PD), along with hemodialysis and kidney transplantation, represents one of the major modalities used in renal replacement therapy that carries the advantages of better preservation of residual renal function and less impact on hemodynamics [1,2]. Because PD is a home-based therapy, it allows patients to maintain their regular lifestyles with minimal disruptions [3]. However, PD may need to be discontinued for various reasons, and PD-related peritonitis represents one of the leading causes of technique failure. In Japan, Gram-positive cocci account for the largest proportion of organisms that cause PD-related peritonitis, at ~39%, although various other organisms have also been implicated [4]. Proper identification of the causative organism and the prompt administration of appropriate antibiotic therapy tailored to the pathogen are essential to the effective management of PD-related peritonitis. In some cases, certain organisms exhibit resistance to treatment, wherein removal of the PD catheter may be deemed necessary. The accumulation of literature detailing clinical experiences concerning the course of PD-related peritonitis when caused by different organisms is therefore crucial to the establishment of optimal treatment strategies for this condition. *Staphylococcus caprae *(*S. caprae*) is a coagulase-negative *Staphylococcus *that is generally considered a skin commensal in humans [5]. Although S. caprae infections have been reported in various organs, cases of PD-related peritonitis caused by *S. caprae* remain rare in the literature. Herein, we report a case of PD-related peritonitis, ultimately identified as being caused by *S. caprae*.

## Case presentation

The patient was an 88-year-old man who had initiated PD three years earlier due to end-stage renal disease secondary to nephrosclerosis. Approximately one month before admission, he developed cloudy PD effluent without fever or abdominal pain. His primary care physician prescribed levofloxacin, which resulted in a transient improvement in effluent clarity; however, the cloudiness recurred, and he was referred to our department for further evaluation. On admission, his vital signs were as follows: body temperature, 36.5°C; blood pressure, 153/82 mmHg; pulse rate, 72/min; and oxygen saturation (SpO₂) 98% on room air. Physical examination revealed no abdominal tenderness, and no signs of infection, such as erythema, pain, or purulent discharge, were observed at the PD catheter exit site or along the subcutaneous tunnel. Laboratory findings on admission were as follows: white blood cell (WBC) count, 4,400/μL with 63.0% neutrophils; blood urea nitrogen, 56 mg/dL; and serum creatinine, 7.40 mg/dL. The WBC count in the PD effluent was elevated to 800/μL, with 83% of the cells being neutrophils (Table 1).

**Table 1:** Laboratory findings on admission WBC: white blood cell; Hb: hemoglobin; Plt: platelet; Alb: albumin; γ-GTP: gamma-glutamyl transpeptidase; AST: aspartate aminotransferase; ALT: alanine aminotransferase; LDH: lactate dehydrogenase; BUN: blood urea nitrogen; Cr: creatine; eGFR: estimated glomerular filtration rate; Na: sodium; K: potassium; Cl: chloride; Ca: calcium; P: phosphorus; CRP: C-reactive protein

Blood test	Value	Reference range
WBC (/μL)	4400	3300-8600
Segmented neutrophils ​​(%)	63	38.0-74.0
Lymphocytes (%)	23.4	16.5-49.5
Monocytes (%)	9.3	2.0-10.0
Eosinophils​​​​​​​ (%)	3.6	0.0-8.5
Basophils​​​​​​​ (%)	0.7	0.0-2.5
Hb (g/dL)	11.3	13.7-16.8
Plt (/mL)	224000	158000-348000
Alb (g/dL)	2.5	4.1-5.1
γ-GTP (U/L)	17	13-64
AST (U/L)	17	13-30
ALT (U/L)	13	Oct-42
LDH (U/L)	162	124-222
BUN (mg/dL)	56	Aug-20
Cr (mg/dL)	7.4	0.65-1.07
eGFR (ml/min/1.73m2)	6.01	>60
Na (mEq/L)	139	138-145
K (mEq/L)	3.5	3.6- 4.8
Cl (mEq/L)	102	101-108
Ca (mg/dL)	8.3	8.8-10.1
P (mg/dL)	3.1	2.7-4.6
CRP (mg/dL)	0.46	0.00-0.14
Peritoneal dialysis effluent test	Value	Reference range
WBC (/μL)	800	<100
Segmented neutrophils (%)	83	-
Lymphocytes​​​​​​​ (%)	15	-
Monocytes (%)	2	-
Eosinophils​​​​​​​ (%)	0	-
Basophils​​​​​​​ (%)	0	-

**Figure 1:**
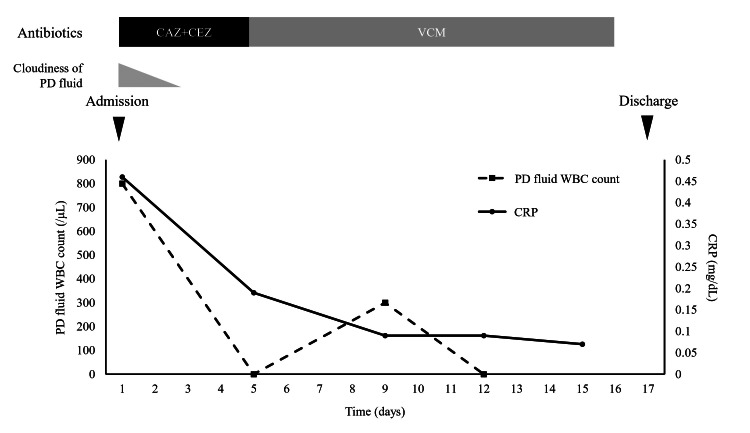
The patient’s clinical course PD: peritoneal dialysis; CAZ: ceftazidime; CEZ: cefazolin; CRP: C-reactive protein; VCM: vancomycin; WBC: white blood cell

Based on these findings, the patient was diagnosed with PD-related peritonitis. He was admitted for treatment and started on intraperitoneal administration of cefazolin (1 g/day) and ceftazidime (1 g/day). After initiating antibiotic therapy, the PD effluent became clear by hospital day 3, and the WBC count in the effluent decreased to 0/μL by hospital day 5 (Figure 1).

On hospital day 6, the PD effluent culture grew *S. caprae*. The culture tests were performed using blood culture bottles. In addition to non-centrifuged samples, cultures were also conducted using samples that had been centrifuged at 4°C, 3000 rpm for 15 minutes, in accordance with the recommendations of the International Society for Peritoneal Dialysis [6]. *S. caprae* was identified in both centrifuged and non-centrifuged specimens. Drug susceptibility testing revealed that the isolate was methicillin-resistant coagulase-negative *Staphylococcus.* Although cefazolin appeared to be clinically effective, the intraperitoneal antibiotic was switched to vancomycin (Table 2). Vancomycin was initially administered at a dose of 1.5 g/day. Thereafter, the dosing was adjusted based on therapeutic drug monitoring (TDM) by measuring serum concentrations. Additional intraperitoneal administrations of 0.5 g vancomycin were given on hospital days 9, 13, and 16.

**Table 2:** Drug susceptibility test results for Staphylococcus caprae MIC: minimum inhibitory concentration; R: resistant; S: sensitive; I: intermediate; N/A: not applicable

Antimicrobial agent	MIC (mg/mL)	MIC interpretations
Oxacillin	>2	R
Sulbactam/Ampicillin	≤2/1	R
Amoxicillin/Clavulanic	≤2/1	R
Cefazolin	≤2	R
Cefoxitin	8	R
Cefmetazole	4	R
Imipenem	≤0.5	R
Meropenem	1	R
Gentamicin	8	I
Amikacin	≤8	N/A
Arbekacin	≤1	N/A
Erythromycin	≤0.25	S
Clindamycin	≤0.5	S
Minocycline	≤1	S
Chloramphenicol	2	S
Vancomycin	≤0.5	S
Teicoplanin	≤0.5	S
Ciprofloxacin	4	R
Levofloxacin	>4	R
Sulfamethoxazole/Trimethoprim	≤1/19	S
Moxifloxacin	>1	R
Linezolid	1	S
Daptomycin	≤0.5	S

In addition, during hospitalization, we reviewed the patient’s PD technique and found that his use of a mask and hand hygiene during the PD fluid exchange was inadequate. Because touch contamination was suspected to be the cause of his PD-related peritonitis, we provided retraining on the proper technique. Thereafter, the PD effluent remained clear without recurrence of cloudiness, and PD-related peritonitis resolved. The patient was discharged on hospital day 17.

## Discussion

We encountered a rare case of PD-related peritonitis caused by *S. caprae*. The patient was successfully treated with antibiotics and did not require PD catheter removal. *S. caprae* is a coagulase-negative *Staphylococcus *species that was first isolated from goat milk in 1983 and is known to colonize the skin and mammary glands of goats [7]. It has subsequently been found to inhabit the skin and nails of healthy humans as well [5]. To date, *S. caprae* has been reported to potentially cause acute otitis externa, urinary tract infections, pneumonia, endocarditis, meningitis, osteoarticular infections, and bacteremia [8]. However, reports on PD-related peritonitis caused by *S. caprae* infection remain limited. Only one previous case of PD-related peritonitis caused by *S. caprae* has been reported in the literature, which was complicated by concurrent infection with *Aspergillus fumigatus* (*A. fumigatus*) as well [9]. To the best of our knowledge, this is the first case report of PD-related peritonitis caused solely by *S. caprae*. The characteristics of our present case, alongside the previously reported one, are summarized in Table 3.

**Table 3:** Cases of PD-related peritonitis caused by Staphylococcus caprae ESRD: end-stage renal disease, PD: peritoneal dialysis

Study	Age/sex	ESRD cause	Duration of peritoneal dialysis	Other bacteria cultured	Clinical findings	Period from onset to treatment (days)	Antibiotic treatment	Catheter removal	Outcome
Engelbrecht et al. [9]	36 years/female	Unknown	Three years	Aspergillus fumigatus	Abdominal pain, cloudy peritoneal fluid	Five days	Vancomycin + amikacin to vancomycin + voriconazole	Yes	Cured
This case	88 years/male	Nephrosclerosis	Nine months	No	Cloudy peritoneal fluid	One month	Cefazolin + ceftazidime to vancomycin	No	Cured

In that prior report, catheter removal was deemed necessary to treat the patient, likely owing to the impact of the concurrent A. fumigatus coinfection. By contrast, our patient responded promptly to antibiotic therapy and achieved remission without requiring catheter removal. Although definitive conclusions cannot be drawn from a single case, our findings suggest that PD-related peritonitis caused by *S. caprae* infection likely carries a favorable prognosis. The major risk factors for *S. caprae* infection have been reported to include immunosuppression, diabetes mellitus, chronic kidney disease, obesity, open fractures, and traumatic bone injuries [10]. In the present case, our patient was at risk because of his advanced age of 88 years, as well as his end-stage renal disease comorbidity that required peritoneal dialysis. Moreover, it became evident during his hospitalization that the patient had not been properly disinfecting his hands during self-administered PD dialysate exchange procedures, indicating nonadherence to aseptic techniques. Given that *S. caprae* colonizes the skin and nails, we believe that touch contamination likely contributed to the infection he developed. Furthermore, in this case, the possibility that *S. caprae* was detected as a contaminant cannot be completely ruled out. However, *S. caprae* was isolated from all specimens, both centrifuged and non-centrifuged, using blood culture bottles. Based on these findings, we believe that *S. caprae* was indeed the causative pathogen of PD-related peritonitis in this case.

Although case reports of PD-related peritonitis caused by *S. caprae* are rare in the literature, previous studies have reported that *S. caprae* accounted for 1.1% (1/93 cases) of PD-related peritonitis in a single-center study, and for 5.9% of cases caused by coagulase-negative Staphylococci (as identified via 16S rRNA gene sequencing) [11,12]. Therefore, despite the paucity of published reports, it is possible that PD-related peritonitis caused by *S. caprae* is more common than it appears. One reason for this low number of reports may be the limitations of certain automated identification systems, which can fail to identify *S. caprae* if their databases do not contain the necessary information [8]. Recently, matrix-assisted laser desorption ionization time-of-flight mass spectrometry (MALDI-TOF MS) has been reported to facilitate the rapid and accurate identification of clinical *S. caprae* isolates [13]. This technique was used at our center to identify the causative organism in the present case. With the increasing use of MALDI-TOF MS and other advanced bacterial identification techniques, the number of reported cases of *S. caprae*-related PD peritonitis may rise in the future.

## Conclusions

We experienced and successfully treated a case of PD-related peritonitis caused by *S. caprae*, which may be associated with a favorable prognosis. With the growing use of advanced identification techniques such as MALDI-TOF mass spectrometry, more cases of PD-related peritonitis due to *S. caprae* are likely to be detected. Despite this, the number of reported cases remains limited. Further accumulation of case reports is needed to validate this favorable clinical course. Additionally, such data will be essential for establishing an optimal treatment strategy for this condition.
